# Granular cell tumor of the appendix: a new case and review of the literature

**DOI:** 10.1186/2193-1801-2-649

**Published:** 2013-12-04

**Authors:** Luca Roncati, Gianrocco Manco, Sebastiano Italia, Giuseppe Barbolini, Antonio Maiorana, Aldo Rossi

**Affiliations:** Department of Diagnostic and Clinical Medicine and of Public Health, Section of Pathology, University of Modena and Reggio Emilia, Struttura Complessa di Anatomia, Istologia e Citologia Patologica, Policlinico Hospital, via del Pozzo 71, 41125 Modena, Italy; Department of Emergency Medicine, Section of Surgery, University of Modena and Reggio Emilia, Modena, Italy

**Keywords:** Granular cell tumor, Ileocecal appendix, Chronic appendicitis, Segmented peritonitis, Oncologic management

## Abstract

Granular cell tumor (GCT) is a rare and usually benign lesion of neural / schwannian origin, most frequently found in middle-age women. The appendicular involvement is extremely rare: in over half a century only twelve cases have been reported in the literature, the patients living in America and Europe. Hitherto, no cases are documented from Africa, Asia and Oceania and no cases of malignant GCTs of the appendix have been reported.

Most patients were diagnosed preoperatively as having acute appendicitis, whereas in three patients the tumor was incidentally detected during major abdominal surgery. The GCTs were equally distributed between mid-appendix and tip, where lymphoid tissue is more abundant and the anatomical nerve supply is progressively reduced. Moreover, the appendix surrounding the GCTs is characterized by the presence of chronic inflammatory cells (histiocytes, plasmocytes, eosinophils, mastocytes) and, therefore, a chronic inflammation of the appendix may be an antecedent condition favouring the appearance of GCTs. The GCT of the appendix appears so to be a lesion that reflects local reactive changes in the neural / schwannian cells, rather than being a genuine neoplasm. We describe the smallest GCT of the appendix ever reported, with a detailed literature review supporting its reactive origin in the lymphatic tissue-rich sites, such as ileo cecal appendix.

## Introduction

Granular cell tumor (GCT) is a rare and usually benign lesion which occurs in different districts of the body, but chiefly in the oral cavity (Vered et al. 2009). It is widely thought to be of neural / schwannian cell origin and it is most often found in middle-age women, with a higher incidence among women of black ethnicity (Rosai 2004; Ordenez 1999). GCT usually presents as a painless, solitary and circumscribed nodule, under 3 cm in diameter, occurring mainly in the tongue, esophagus, skin, muscle or subcutaneous tissue (Weiss 2007; Zoccali et al. 2011; Lack et al. 1980). However, it can also appear in internal organs, involving the respiratory or urinary tract and the central nervous system. The tumor may be multiple (in 10-15% of cases) (Weiss 2007), particularly in black patients. Its location in the gastrointestinal tract (gt) is rare (5%) (Zoccali et al. 2011). The presence of gtGCT, which manifests as a circumscribed submucosal nodule, is often detected incidentally during endoscopy or surgical resection. Many of the gtGCTs do not exceed 2 cm in diameter and the tumor does not infiltrate the muscularis propria. The appendix is rarely affected; in more than half a century (from 1956 (Wanick 1956) to the present) only twelve cases have been reported in medical literature (Zoccali et al. 2011; Wanick 1956; Hausman 1963; Apisarnthanarax 1981; Sarma et al. 1984; Fried et al. 1984; Pipeleers-Marichal et al. 1990; Kaltschmidt et al. 1992; Gavelli et al. 2005; Moreno Gijon et al. 2009; Saleh et al. 2009; Singhi & Montgomery 2010). We describe the smallest GCT of the appendix ever reported, with a detailed literature review supporting for the first time its reactive origin in the lymphatic tissue-rich sites, such as ileo cecal appendix.

## Case description

A 49-year-old woman, who had been suffering for some time from irritable bowel syndrome (IBS), presented to the emergency department complaining of pain in the right lower quadrant (RLQ). She reported a two-week history of constipation and pain in the RLQ and a temperature of 38.5°C. Over the last two days, she had experienced pain at defecation, tenesmus, rectal bleeding and episodes of hematuria. The condition showed no improvement with antispastic therapy or pain killers. Her medical history included IUD insertion/retrieval and two uncomplicated deliveries, while her family history included colonic adenocarcinoma in her mother. The blood test pointed out leukocytosis and elevated concentration of CRP.

**Figure 1:**
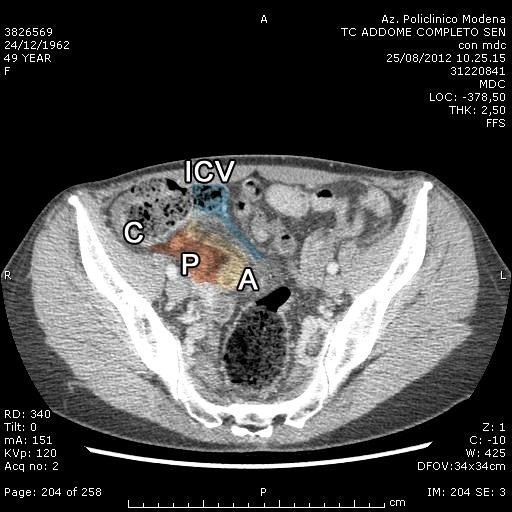
**CT scan with contrast medium showing a bent phlegmonous appendix with a swollen tip enclosed by an inflammatory plastron tenaciously adherent to the last ileal loop and to the right fallopian tube fimbria [A = appendix; C = caecum; ICV = ileo-caecal valve; P = plastron].**

At the medical examination a flat, sore abdomen in the RLQ and the presence of a deep stiff mass with undefined borders were observed. CT scan pointed out an appendix rising postero-medially to lumbosacral level for about 10 cm; it was markedly thickened and patchy, with increased density of the surrounding fat tissue consistent with a phlegmon (Figure 1). Periureteral tissue was affected by the inflammatory reaction. Abdomino-pelvic lymph nodes were slightly enlarged, in particular at the mesenteric periappendicular level, the axial size rising to 20 mm. Given the clinical presentation and radiological findings, the patient underwent a diagnostic laparoscopy. A bent, phlegmonous appendix, 6.5 cm in length and with a swollen tip, was noted enclosed within an inflammatory plastron; it adhered tenaciously to the last ileal loop (at the caecal fundus) and to the right fallopian tube fimbria. A laparoscopic appendectomy was performed with a careful management, isolating the appendix and separating it from the tube and the thickened last ileal loop. A stiff greyish right ovarian lesion of 3.7 × 3.8 cm was also found; the left ovary had no significant macroscopic changes.

The surgical specimens were fixed in 10% neutral buffered formalin and then paraffin embedded. Besides to haematoxylin-eosin, histochemistry (Luxol fast blue, Pas-diastase) and immunohistochemistry (Ki-67, p53, S100, NSE, calretinin) were performed, applying the standard avidin-biotin complex (ABC) method.

**Figure 2:**
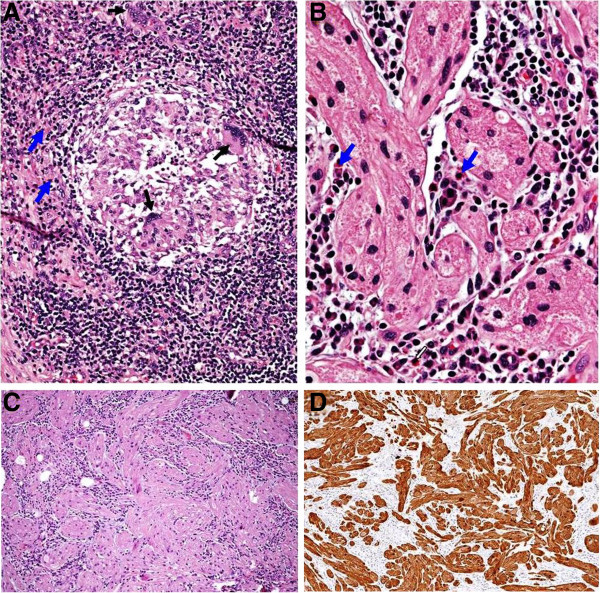
**Tip of the appendix.** Chronic granulomatous inflammation adjoining submucosal GCT is noticeable. Foreign body-type multinucleated giant cells (black arrows), mastocytes and eosinophils (blue arrows) are seen together with lymphocytes and plasmocytes (**A** and **B**, hematoxylin-eosin, original magnification x4 and x10). The round, oval, spindle-shaped tumor cells display a granular cytoplasm (**C**, hematoxylin-eosin, original magnification x4), strongly immunoreactive for S-100 protein (**D**, original magnification x4).

**Figure 3:**
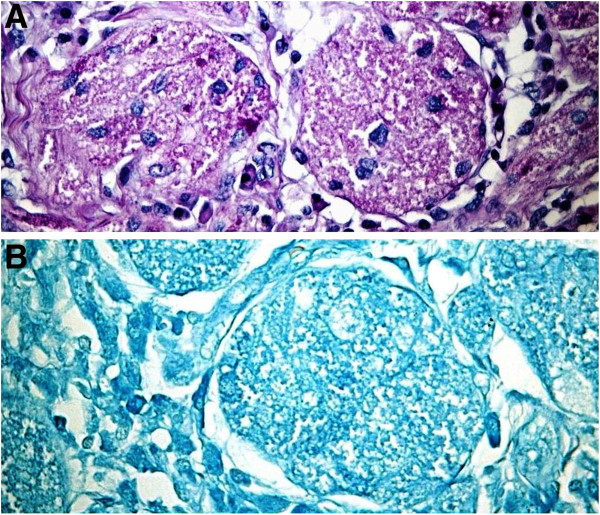
**The tumor granules are stained by PAS-diastase (A, original magnification x40) and Luxol fast blue (B, original magnification x40).**

**Figure 4:**
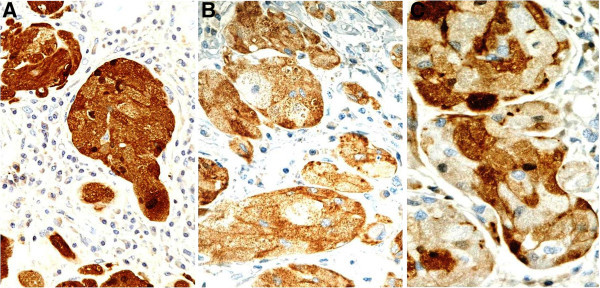
**The tumor granules are immunoreactive for S-100 protein (A, original magnification x40), NSE (B, original magnification x40) and calretinin (C, original magnification x40) with a variable range of intensity.** The tumor cell nests are surrounded by plasmocytes and lymphocytes.

Specimens examination revealed a florid granulomatous chronic inflammation with foreign body-type multinucleated giant cells and mastocytes (Figure 2) in the swollen tip of the appendix. Adjacent to this, a submucosal GCT nodule was present, 0.2 cm in diameter (Figures 2, 3 and 4). The tumor was uncapsulated, well circumscribed and showed no trend to infiltration; it was devoid of necrosis and cytological atypia and showed a low cytoproliferative activity (MIB1-LI). The tumor cells resulted immunoreactive for S-100 protein, neuron-specific enolase (NSE) and calretinin; p53 was not detected. The cytoplasmatic granules were ascertained by Luxol fast blue and Pas-diastase stainings. Above the tip, the phlogosis was acutely exacerbated. There was a prevalence of neutrophils and fibrin in relation to a perforation of the appendix wall and a reactive septated peritonitis. The ovarian specimen proved to be a classic leiomyoma (fibroma) without cell atypia; the fallopian tubes were slightly atrophic. The postoperative phase was uneventful and the patient was discharged on postoperative day four. An integrated PET-CT scan performed one month after hospital discharge revealed no signs of recurrence. In view of the histopathological diagnosis of benignancy and family history of colonic cancer, the oncologist suggested a follow-up with colonoscopy every 3 years. In literature no data are reported, which correlate the GCT incidence with the risk for colorectal cancer, and our effort has been aimed in the clinical management of patients affected by GCT involving the lymphatic tissue-rich sites, such as ileo cecal appendix.

## Discussion

GCT was first described in 1854 by Weber (Weber & Virchow 1854) as a cluster of large cells featuring granular eosinophilic cytoplasm. In 1926, Abrikosoff (Abrikossoff 1926) named these lesions granular cell myoblastomas and he assumed, observing a group of five GCTs located on the tongue, that GCT had a striated muscle origin. In 1952, Feyrter (Feyrter 1952) renamed this form of tumors ‘granular cell neuromas’ , pointing out the tendency of such lesions to affect peripheral nerves and presuming a perineural origin. Other obsolete terms for this tumor were granular cell neurofibroma and granular cell schwannoma (Feyrter 1952). At the present time, the term granular cell tumor is worldwide used, even if (as Vered *et al. s*uggest (Vered et al. 2009)) GCT might be considered as lesion that reflect a local metabolic or reactive change rather than a true neoplasm. Rosai (Rosai 2004) highlights further that focal clusters of granular cells, analogous to GCT cells, can occur in a variety of neoplastic and non neoplastic conditions, whereas to qualify for GCT diagnosis, the entire lesion has to be granular. GCT is a benign lesion characterized by the presence of plump cells often crowded together with abundant granular cytoplasm. The cell borders may appear indistinct, making it resemble a syncytium. Its benign status is further attested by the fact that no recurrences are reported, not even in lesions whose excision is incomplete (Vered et al. 2009). Histomorphological reports suggest that the granular cells may also be round, oval, polygonal or spindle-shaped and that the nuclei may be dark or vesicular, being located in variable positions within the cell (Vered et al. 2009). The eosinophilic cytoplasm presents fine-to-coarse granularity. The phagolysosome granules contain large amounts of hydrolytic enzymes (such as acid phosphatase) and are strongly PAS-positive, diastase-resistant and consistently positive for Luxol fast blue and myelin basic protein. The tumor cells are immunoreactive for S-100 proteins, calretinin, NSE, laminin and CD68 (Kp1) (Vered et al. 2009; Rosai 2004; Weiss 2007), but they do not react with antibodies for neurofilaments or glial fibrillary acidic protein (GFAP) (Rosai 2004; Ordenez 1999; Weiss 2007). The nerve supply tapers gradually from the base to the tip of the appendix where it is consequently more liable to damage from reactive changes and local inflammation. Nowadays, no case of GCT was reported at the base of the appendix. Embryologically, the appendix develops as part of the mid gut. In humans, it is a vestigial organ with glands forming simple tubes, which are often forked and secrete from 1 to 2 ml of mucinous fluid daily. Lymphoid nodules are also present, these being abundant and confluent in the mid-appendix and particularly at the tip (Sams 1992). The amount of lymphoid tissue varies with age: there is little in the foetal appendix, while it increases after birth and attains its maximum at puberty, thereafter gradually declining. During late middle life, little lymphoid tissue remains (Morson 1966). This sequence of growth and atrophy of the appendiceal lymphoid tissue correlates with the age incidence of acute appendicitis, which is prevalent in young patients, and with the site of GCT occurrence.

## Conclusion

The present case is the smallest (2 mm) GCT of the appendix ever reported in literature. For its small size below the imaging resolution capability, it was an incidental finding in the course of a histopathological examination for acute appendicitis.

A detailed analysis (Table 1) of the features of GCTs specific to the appendix reveals that the cases so far quoted in the literature were found in the New World (USA and Brasil) and in Europe (Belgium, Germany, Principality of Monaco, Spain and Italy). There was no prevalence among black ethnicities, whereas no cases were reported from Africa, Asia and Oceania. Moreover, as Gavelli *et al*. (Gavelli et al. 2005) have pointed out, no cases of malignant GCT in the appendix have been detected. On the other hand - comparing the features of GCTs in general with those specific to the appendix- the M/F ratio (1:1.1 versus 1:1.3) and the mean age (41.6 versus 40.0) are in a very similar value range. Most patients were admitted to the emergency department with a pre-operative diagnosis of acute appendicitis, whereas, in three patients, the tumor was an incidental finding during major surgery to the abdomen. The GCT location was equally distributed between mid-appendix and tip, in keeping with the anatomical nerve supply. In our case, a personal history of IBD supports the hypothesis that the acute exacerbation of chronic appendicitis is due to a marked modification of the gut microbiota (Doré & Corthier 2010; Serino et al. 2012), an event which is well-attested in the course of IBD (Belmonte et al. 2012). From the foregoing, we can deduce that a chronic inflammation, evidenced by the presence of histiocytes, eosinophilis, plasmocytes and mastocytes surrounding the GCTs, is an antecedent condition favouring the appearance of GCTs in the appendix and that the GCT in the appendix is a lesion which reflects local reactive changes of the neural / schwannian cells rather than a true neoplasm. This finding is congruent with the data reported by Vered *et al.* (Vered et al. 2009) for GCTs in the oral cavity.

**Table 1:** **Cases of granular cell tumors (GCTs) of the appendix reported in literature**

Case number	Author(s) year	Sex age	Country race	Appendix length	Tumor location	Tumor diameter	Tumor nodule	Surrounding appendix	Concomitant pathology
**1**	Wanick 1956	F	Brasil	NR	Mid-	1 cm	Double	Acutely exacerbated chronic appendicitis	None
		34	NR		appendix	0.5 cm			
**2**	Hausman 1963	M	USA	3.5 cm	Tip	0.8 cm	Single	Acute appendicitis	None
		45	Caucasian						
**3**	Apisarnthanarax 1981	F	USA	NR	NR	5 cm	Single	NR	NR
		34	Caucasian						
**4**	Sarma et al. 1984	M	USA	6 cm	Mid-appendix	0.5 cm	Single	Acute appendicitis with abdominal sepsis and subphrenic abscess	Small bowel obstruction, diverticulitis of the transverse colon with perforation
		58	African						
**5**	Fried et al. 1984	F	USA	NR	Mid-appendix	0.8 cm	Single	No evidence of inflammation	Multifocal GCT of the GI tract (esophagus, stomach, cecum)
		38	African						
**6**	Pipeleers-Marichal et al. 1990	M	Belgium	9 cm	Tip	4 cm	Single	Chronic appendicitis and neuroma from radiation injury	Rectal adenocarcinoma
		47	Caucasian						
**7**	Kaltschmidt et al. 1992	M	Germany	7 cm	NR	NR	Single	Acute appendicitis	None
		32	Caucasian						
**8**	Gavelli et al. 2005	M	Principality of Monaco	5 cm	NR	0.5 cm	Single	Acute suppurative appendicitis	None
		46	African						
**9**	Moreno Gijon et al. 2009	F	Spain	NR	Tip	1 cm	Single	NR	None
		33	NR						
**10**	Saleh et al. 2009	F	USA	NR	NR	NR	Single	NR	Multifocal GCT of the GI tract (colon, mesentery) and recto-sigmoid mass
		62	Caucasian						
**11**	Singhi and Montgomery 2010	F	USA	NR	NR	0.6 cm	Single	Acute appendicits	None
		45	Caucasian						
**12**	Zoccali et al. 2011	M	USA	13 cm	Mid-appendix	3.5 cm	Single	Acute appendicitis	None
		19	Caucasian						
**13**	Roncati L et al. 2013	F	Italy	6.5 cm	Tip	0.2 cm	Single	Acutely exacerbated chronic appendicits	Right ovarian fibroma
		49	Caucasian						

NR = not reported.
